# A first-in-human randomized, double-blind, placebo-controlled, single- and multiple-ascending oral dose study of novel spiroindolone KAE609, to assess the safety, tolerability and pharmacokinetics in healthy adult volunteers

**DOI:** 10.1186/1475-2875-13-S1-O37

**Published:** 2014-09-22

**Authors:** Joel Leong, Ruobing Li, Jay Prakash Jain, Gilbert Lefèvre, Baldur Magnusson, Thierry Diagana, Peter Pertel

**Affiliations:** 1 Novartis Institute for Tropical Diseases, Singapore; 2 Novartis Institute of Biomedical Research, Beijing, China; 3 Novartis Healthcare Private Limited, Hyderabad, India; 4 Novartis Pharma AG, Basel, Switzerland; 5 Novartis Institutes of Biomedical Research, Cambridge, USA

## Background

New antimalarials are needed to address the threat of emerging artemisinin resistance. KAE609 is a spirotetrahydro-β-carbolines (spiroindolone) derivative and the first molecule of this new class entering phase II studies.

## Materials and methods

This first in human randomized, double-blind, placebo-controlled, ascending single and multiple oral dose study was designed to evaluate the safety, tolerability and pharmacokinetics of KAE609 in healthy volunteers. The study comprised of single ascending dose (1-300 mg, including one 30 mg food effect cohort, with 4-10 subjects in each cohort) and multiple ascending dose (10-150 mg once daily for 3 days with 8 subjects in each cohort) cohorts. The follow-up period was 6-8 days post last dose. Safety and pharmacokinetics were assessed at scheduled time points during the study.

## Results

Of the total 95 male subjects enrolled, 93 completed the study. Participants in both cohorts had comparable demographics. Systemic exposure in terms of AUC_inf_ increased in a dose-proportional manner over the dose range of 1-300 mg. AUC_last_ and C_max_ also increased in an approximately dose-proportional manner in single and multiple dose cohorts respectively, though not considered statistically dose-proportional. When administered daily for three days, the accumulation ratio on Day 3 (AUC_0-24_, day 3/AUC_0-24_, day 1) was in the range of 1.5-2.0 in the studied dose range (10-150 mg) and consistent with an elimination half-life of around 24 hours. Urine analysis for unchanged KAE609 revealed negligible amounts (≤0.01%) excreted renally. The high fat food intake with 30 mg single dose did not affect the extent of KAE609 absorption however C_max_ was reduced by around 27% and increased T_max_ (from 2.5 to 4 hours). KAE609 was generally well tolerated within this study, with transient gastrointestinal and genitourinary adverse events of mild to moderate intensity, which increased with rising doses.

## Conclusions

KAE609 is a new antimalarial with potential to address artemisinin resistance, and demonstrates a half-life of around 24 hours and dose-proportional increase in systemic exposure. KAE609 was generally well tolerated within this study, with mild to moderate intensity gastrointestinal and genitourinary adverse events reported with increasing dose. There were no study findings that would preclude dosing in malaria patients and a human malaria patient trial, which immediately followed this study, showed that KAE609 clears parasitemia rapidly in *falciparum* and *vivax* malaria patients.

